# A Semi-Supervised 3D Indoor Localization Using Multi-Kernel Learning for WiFi Networks

**DOI:** 10.3390/s22030776

**Published:** 2022-01-20

**Authors:** Yuh-Shyan Chen, Chih-Shun Hsu, Ren-Shao Chung

**Affiliations:** 1 Department of Computer Science and Information Engineering, National Taipei University, No. 151, University Rd., San Shia District, New Taipei City 237, Taiwan; yschen@mail.ntpu.edu.tw (Y.-S.C.); boredconversation@gmail.com (R.-S.C.); 2 Department of Information Management, Shih Hsin University, No. 1, Ln. 17, Sec. 1, Muzha Rd., Wenshan District, Taipei City 116, Taiwan

**Keywords:** 3D indoor localization, multi-kernel, WiFi, transfer learning, semi-supervised learning

## Abstract

Indoor localization is an important issue for indoor location-based services. As opposed to the other indoor localization approaches, the radio frequency (RF) based approaches are low-energy solutions with simple implementation. The kernel learning has been used for the RF-based indoor localization in 2D environment. However, the kernel learning has not been used in 3D environment. Hence, this paper proposes a multi-kernel learning scheme for 3D indoor localization. Based on the signals collected in the area of interest, the WiFi signals with better quality and closer to the user are selected so as to reduce the multipath effect and the external interference. Through the construction of multi-kernel, the localization accuracy can be improved as opposed to the localization based on the single kernel. We build multiple kernels to get the user’s location by collecting wireless received signal strengths (RSS) and signal-to-noise ratios (SNR). The kernel learning maps data to high dimension space and uses the optimization process to find the surface where the data are mapped. By multi-kernel training, the surface is fine-tuned and eventually converges to form the location database during the mapping process. The proposed localization scheme is verified by the real RSS and SNR collected from multiple wireless access points (AP) in a building. The experimental results verify that the proposed multi-kernel learning scheme performs better than the multi-DNN scheme and the existing kernel-based localization schemes in terms of localization accuracy and error in 3D indoor environment.

## 1. Introduction

Recently, the location-based service (LBS) for internet of things (IoT) becomes popular in many fields such as office building, shopping mall, and community building. Since the GPS system cannot catch satellite signals in an indoor environment, many indoor localization systems based on different RF signals have been presented, such as RFID [1,2,3], Bluetooth, and LoRa [4] based localization systems.

With the popularity of wireless LANs and wireless access devices, WiFi-based indoor localization has become more and more popular in recent years. Based on the WiFi localization systems, fingerprint schemes [5,6,7,8,9,10] show great advantages and accuracy in indoor localization with RSS and SNR signals. In the offline phase, select multiple physical locations in the region of interest and the signals received from APs built in indoor environment are defined as labeled data in these selected locations. Therefore, the obtained position and the corresponding labeled data are defined as the fingerprint database. In the training phase, the labeled data and the corresponding RSS and SNR are obtained. In the online phase, the RSS and SNR received by devices determine the specific location based on the radio map. Consequently, this kind of scheme shows high potential in indoor localization.

A few 3D indoor localization schemes have been proposed. Qi et al. [11] proposed a hybrid RSS-AOA measurement in non-cooperative and cooperative 3D wireless sensor networks. By using approximate error expressions for the RSS and angle of arrival (AOA) measurement models, new estimation based on the proposed method can be obtained. Cramariuc et al. [12] proposed a Penalized Logarithmic Gaussian Distance metric, which can improve the comparability of different clustering schemes in 3D positioning.

The existing localization schemes either need additional hardware and measurements or need to collect numerous samples for learning or training. To reduce the costs of hardware, collecting, and measurements, a novel semi-supervised localization scheme is proposed, which only needs to collect a few labeled signals of RSS and SNR and hence reduces the costs (only 25% of the collected signals are labeled). However, the accuracy of the existing localization schemes using only a few labeled signals of RSS and SNR are not satisfactory. To improve these schemes, the multi-kernel learning scheme is proposed and adopted in our semi-supervised localization scheme so as to improve the localization accuracy and alleviate the multipath effect and signal deviating problem. The kernel model is adopted because the kernel model can learn the distribution of data according to the kernel function selected for the problem, and judge the result according to the distribution of the data. During localization, the kernel model can learn the distribution of signals in the positioning area, and determine where the user may be based on the signal distribution.

The main contributions of the proposed approach are summarized as follow:

- A semi-supervised localization scheme, which only needs to collect a few labeled signals of RSS and SNR, is proposed in this paper.
- A multi-kernel learning scheme with weight adjustment and optimization for 3D indoor localization in WiFi networks is proposed in this paper so as to further improve the accuracy of localization.
- Experimental results demonstrate that the proposed localization scheme performs better than the multi-DNN scheme and the existing kernel-based localization schemes in terms of localization accuracy and error.

The rest of this paper is organized as follows. Section 2 describes the related works. Section 3 describes the system model and the problem formulation of the proposed scheme. Section 4 describes the proposed semi-supervised multi-kernel learning scheme for 3D indoor localization. The experimental results are shown in Section 5, and the conclusions are summarized in Section 6.

## 2. Related Work

This section mainly describes the related work and discusses the motivation.

### 2.1. Traditional RSSI-Based Localization Schemes

Zanca et al. [13] compared the experimental results of several traditional RSSI-based localization schemes for indoor wireless sensor networks. Among these traditional RSSI-based localization schemes, i.e., Min–Max, Multilateration, Maximum Likelihood (ML), and ROCRSSI, the ML scheme performs the best. The ML scheme is based on classical statistical inference theory. In testbed 1, the localization error of the ML scheme is around 2.3 m where there are 25 anchor nodes located in a 10×10 m${}^{2}$ room. In testbed 2, the localization error of the ML scheme is around 1.3 m where there are 40 anchor nodes located in a 7×7 m${}^{2}$ room. However, a mobile beacon node that is assumed to be always aware of its own position is required in testbed 2. The assumptions of these traditional RSSI-based localization schemes are different from those of the AI-based localization schemes. Besides, the performance of these traditional RSSI-based localization schemes is not better than that of the AI-based localization schemes. Hence, the traditional RSSI-based localization schemes are not compared with the proposed localization scheme.

### 2.2. Fingerprint-Based Localization

There are lots of localization results [5,7,8,9,10] based on the fingerprint approach which are described as follows. Abbas et al. [5] proposed a WiDeep system, which is a system based on deep learning to achieve fine-grained and powerful accuracy in the presence of noise. This scheme uses a deep learning model of a stacked denosing auto encoder and a probability architecture to process the inference for the received WiFi signal and find the relationship between the WiFi signal heard by the mobile device and its position. Anzum et al. [6] proposed a zone-based indoor positioning method using counter propagation neural networks (CPN). When the traditional CPN is applied, many empty clusters are generated. A slight modification of the CPN can significantly reduce the number of empty clusters and provide more accurate results. Chang et al. [7] proposed a high-precision indoor localization scheme based on WiFi fingerprint. This scheme requires only a single WiFi AP and a single fixed-location receiver. By training DNN based classification model with CSI, it can localize the target without any attached device. Wu et al. [14] proposed a DNN-based fingerprint recognition method called DNNFI. The proposed method only maintains a single DNN between different reference points instead of using multiple deep self-encoders, so it has faster inference calculations and reduces the weight deviation. First, a bunch of auto-encoders are used to pre-train weights. The softmax function is used to determine the probability of the target’s location, which can ultimately be used to estimate the receiver’s location. Although the fingerprint approach has many advantages, including high accuracy and simple implementation, it requires a lot of training data and manual labelling. When the localization area is too large, the fingerprint approach will not be suitable.

### 2.3. Kernel-Based Localization

Kernel learning combining with support vector machine (SVM) paradigm leads to the breakthroughs in many artificial intelligence tasks and gives birth to the kernel learning as a field of research. Yan et al. [15] proposed a people counting approach based on fingerprinting localization for multiple regions in indoor environment. This method locates each target in the environment, and uses the Kernel Fuzzy C-Means (KFCM) clustering algorithm to divide the entire area into different sub-areas to obtain the number of people in each sub-areas. The position is compared with the boundaries of all subregions to determine the subregion to which it belongs. Mari et al. [16] proposed a kernel online sequential extreme learning machine (KOS-ELM) based approach. The proposed method incorporates both trilateration and fingerprinting algorithms in localization phase to improve the localization accuracy. Zou et al. [17] proposed a WinSMS System. The system extracts real-time RSS readings between routers to make WiFi routers as online reference point. Using online data from offline calibration radio maps and labeled source data, RSS readings from the target mobile device are combined as unlabeled data to use transfer learning algorithms to design localized models. The proposed method can learn the invariant kernel by calculating the source distributions and target distributions for reproducing Hilbert kernel space.

### 2.4. 3D Localziation

Among the 3D localization schemes, some schemes are fully 3D [3,11,18,19], some schemes only indicate the floor and the 2D location of the target object [12,20].

Qi et al. [11] proposed hybrid RSS (received signal strength) and AOA (angle-of-arrival) estimators based on the least squares (LS) criterion for non-cooperative and cooperative 3D wireless sensor networks. By applying the convex relaxation techniques, estimators can be transformed into mixed semi-definite programming (SDP) and second-order cone programming (SOCP) problems. Theoretical analysis and simulation results show that the proposed hybrid RSS-AOA estimators can achieve lower Root Mean Square Error (RMSE). Wen et al. [18] proposed an efficient approximate maximum likelihood algorithm, which updates the direction-of-arrival (DOA) and time delay (TD) parameters alternatively. The approximate maximum likelihood algorithm applies to arbitrarily distributed arrays. Then, the author proposes a closed Cramer–Rao boundary for joint DOA and TD estimation. On this basis, the authors provide further analysis to show the benefits of joint DOA and TD estimation over only DOA estimation. Wang et al. [19] proposed a 3D indoor localization algorithm named LMR (LLS-Minimum-Residual) to improve the accuracy of localization. The NLOS (non-line of sight) error is estimated and used to correct the measurement distances. The target location is calculated with the linear least squares (LLS) solution. The final location is obtained by NLOS error mitigation. Cheng et al. [3] proposed a 3D localization scheme (3DLRA) based on deep learning. 3DLRA combines the absolute positioning and relative positioning technology of RFID (Radio Frequency Identification). Through analyzing the variation characteristics of the received signal strength (RSSI) and Phase and mining data characteristics by deep learning, the location accuracy and system stability are improved.

Cramariuc et al. [12] proposed a Penalized Logarithmic Gaussian Distance metric which can enhance the performance of both 3D and RSS clustering. Marques et al. [20] proposed a fingerprint-based indoor localization scheme which estimates the building, then the floor, the room and, finally, the most probable geometric position within the room where the user is located. The positioning process started with the construction of a Filtered Radio Map where the Test Sample is used as a key. Then the similarity between the Test Sample and each one of the Calibration Samples in the Filtered Radio Map is computed. The computation cost is reduced by the filtering stage. Based on filtering stages and majority rules, the estimated building, floor and room are derived. By computing the centroid of the most similar Calibration Samples from the estimated room, the estimated geometric position is obtained. The average localization error is 3.351 m.

### 2.5. Motivation

The existing 3D localization schemes either need additional hardware and measurements or need to collect numerous samples for learning or training. To reduce the cost, we propose a semi-supervised localization scheme for WiFi networks. Although WiFi-based fingerprint and kernel learning schemes show good accuracy in a 2D scenario. However, these approaches may inaccurately detect the floor when the device is located in a 3D environment since it is difficult to distinguish RSS from APs with different distances on different floors. Even if by direction-of-arrival and time delay to estimate the location in 3D environment, the WiFi signal strength is relatively susceptible in 3D environment due to its multipath effect and external interference which lead to inaccuracy. To improve the existing WiFi-based fingerprint and kernel learning schemes, an iterative multi-kernel learning scheme for 3D indoor localization in WiFi networks is proposed in this paper.

Our goal is to improve the localization accuracy in 3D indoor environments by using WiFi signals. The proposed 3D indoor localization scheme can effectively reduce the WiFi indoor positioning error with a small number of labeled data sets, and integrate the existing kernel learning through the optimization fine-tuning method to improve the localization accuracy. The proposed kernel learning method can learn the relationship between the labeled data and unlabeled data based on cross-domain data, which represent the correspondence between the labeled data and unlabeled data.

## 3. Preliminaries

This section introduces the system architecture, problem formulation, and the comparison with the single kernel scheme.

### 3.1. System Architecture

**Figure 1:**
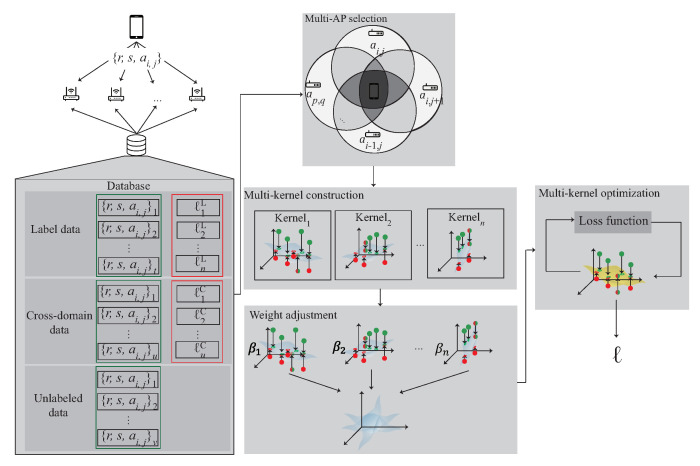
System architecture.

The WiFi 3D indoor localization architecture is shown in Figure 1. There are many APs deployed in the 3D indoor environment with multiple floors. The mobile devices are placed in the localization area. The mobile device can retrieve the RSSI, SNR and the corresponding MAC address within the transmission range of the AP as the identifier. Let *D* denote the data which is retrieved by a mobile device on the location *ℓ*. *D* can be represented as $D=(\left(r_{1},s_{1},m_{1}\right),\left(r_{2},s_{2},m_{2}\right),\ldots ,\left(r_{n},s_{n},m_{n}\right),\ell )$, where r,s,m denote RSSI, SNR and MAC address, respectively, *n* is the number of APs which can be heard by the mobile device within the transmission range. All the data are saved in MongoDB for the training phase.

When the dataset *D* is collected, the next step is to categorize the dataset into two categories, labeled data and unlabeled data based on whether or not the data are collected at known locations. In the training phase, each labeled data from the source domain is correspond to the true location *ℓ*. In order to find the mapping relationship between the labeled data and unlabeled data, the kernel optimization learning is used to form the location database. Each kernel learns the distribution of its own region. In the localization stage, the signal distribution obtained by the mobile device is sent to the server. The server then calculates the expected value of the related kernels and obtains the estimated location. Note that, the training, learning, and location estimation are all performed in the server.

In the proposed scheme, an anchor point located at the corner of the building, whose latitude, longitude, and altitude have been obtained through GPS, is regarded as the point of origin (0, 0, 0) in our coordinate system. The Cartesian coordinate system is adopted in the proposed scheme and a meter is regarded as the basic unit in our coordinate system. We get the estimated relative coordinate (*x*, *y*, *z*) of the target object in our coordinate system first and then we use the anchor point’s position to derive the target object’s estimated location. The reason the GPS coordinates are used because we can use the relative coordinate of the target object to get the possible latitude, longitude, and altitude of the target object. We get the relative coordinate of the labeled data by combining the blueprint of the building and a distance measuring instrument (e.g., laser ranger or ruler). The measuring error is less than 10 cm. To get the relative coordinates of all the labeled nodes indeed consumes a lot of manpower. Hence, we propose a semi-supervised localization scheme.

**Figure 2:**
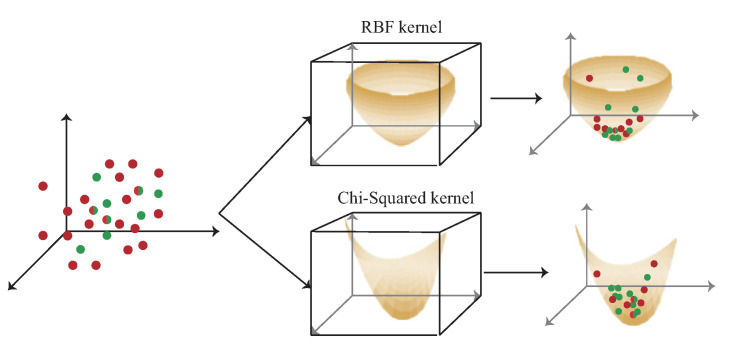
The comparison with RBF kernel and chi-squared kernel.

Figure 2 shows the difference between the RBF kernel and the chi-squared kernel. The data are mapped to high dimension by RBF kernel and chi-squared kernel. In RBF kernel, the signal obtained by users with short distance movement is not obviously different after mapped to high-dimension by RBF kernel which leads to higher localization error as opposed to the chi-squared kernel. Hence, the chi-squared kernel is adopted in the proposed multi-kernel learning scheme for 3D indoor localization.

### 3.2. Problem Formulation

Transfer kernel learning [15,17] has been studied widely in recent years and has been applied successfully in indoor localization. However, when the location field is too large resulting in lots of data groups, the accuracy of transfer kernel learning is decreased and becomes unsuitable. Therefore, the multi-kernel optimization learning method is proposed to solve the above problem.

Assume that *p* APs are installed in an indoor environment, and the signals received by mobile devices from these APs in three dimension position *ℓ* can be represented by a matrix *X*, which includes *q* samples. The received signal $x_{i}=\left(r_{i},s_{i}\right)$ represents the *i*-th RSSI and SNR, respectively. *X* can be represented as $X=[x_{1},x_{2},\ldots ,x_{q}]$.

The signal collected at the offline calibration is named as the source domain, which is denoted as $\left(x_{i}^{s},l_{i}^{s}\right)$. The signal collected in the online localization is named as the target domain and is denoted as $\left(x_{i}^{T}\right)$, which is the data needed to be estimated its location. The goal of the proposed scheme is to estimate the location ($\ell_{T}$) of mobile device by constructing a localization model using multi-kernel optimization learning.

Multi-kernel optimization learning improves the accuracy based on labeled data. There are two domains $D_{S}$ and $D_{T}$, where $D_{S}={\left\{x_{i}^{S},\ell_{i}^{S}\right\}}_{i=1}^{q}$ indicates the data in the source domain and the corresponding labeled data $\ell_{i}$, and the target domain $D_{T}={\left\{x_{i}^{T}\right\}}_{i=1}^{n}$ indicates the unlabeled data. In order to find the relationship between the source domain and the target domain, the cross domain is used. The cross domain is the intersection with the source domain and the target domain and can be represented as $D_{ST}\in D_{S}\cap D_{T}$. By using the cross domain data, the extrapolated kernel is built. The detailed methodology of building extrapolated kernel is introduced in the next section. During the training phase, in order to minimize the difference between the source domain and the target domain, the squared loss optimization problem can be used, which is shown as follows:

(1) $$\begin{array}{c} \underset{\Lambda }{\arg\min^{*}}\left(\;\bar{\;K\;}{\;}_{M}-K_{M}\right)=\arg\min^{*}\left(\;\bar{\;\Phi \;}{\;}_{S_{i}}\Lambda_{T_{i}}\Phi_{S_{i}}^{T}-K_{M}\right).\\ \;subject\;to\left\{\begin{array}{c} \Lambda_{T}=diag\left\{\lambda_{1},\lambda_{2},\cdots ,\lambda_{l}\right\}\\ \;\lambda^{T}Q_{i}\lambda -2r_{i}^{T}\lambda +s_{i}\geq 0,i\in Z^{+}\\ \;\lambda_{i}\geq 0 \end{array}\right.\\ \; \end{array}$$

where $K_{M}$ denotes the source domain $D_{S}$ mapped to high dimension through the kernel function and $\;\bar{\;K\;}{\;}_{M}$ represents the hyperplane found by Mercer’s theorem, $\;\bar{\;\Phi \;}{\;}_{M}$ is the eigenvector matrix of $\;\bar{\;K\;}{\;}_{M}$ and $\;\bar{\;\Phi \;}{\;}_{M}^{T}$ is the transpose matrix of $\;\bar{\;\Phi \;}{\;}_{M}$. The restriction condition is defined as the quadratic constrained quadratic programming (QCQP) optimization problem.

The QCQP is an optimization problem in which both the objective function and the constraints are quadratic functions. It has the following form.

(2) $$\begin{array}{c} \min(\frac{1}{2}x^{T}P_{0}x+q_{0}^{T}x).\\ \;\\ subject\;to\left\{\begin{array}{c} \frac{1}{2}x^{T}P_{i}x+q_{i}^{T}x+r_{i}\leq 0,i=1,2,\cdots ,m\\ \;Ax=b \end{array}\right.\\ \; \end{array}$$

where $P_{0},P_{1},\cdots ,P_{m}$ are *n*-by-*n* matrices, $q_{0},q_{1},\cdots ,q_{m}$ are positive semidefinite matrices and $x\in R^{n}$ is the optimization variable.

The variable Λ is defined as the eigenvalue in the training phase. The purpose is to compute the domain-invariant kernel $K_{A}$ and estimate the location of mobile device. The goal of the above equation is to minimize the difference of the domain location error between the source and target domain in raw data.

### 3.3. Comparison with the Single Kernel Scheme

**Figure 3:**
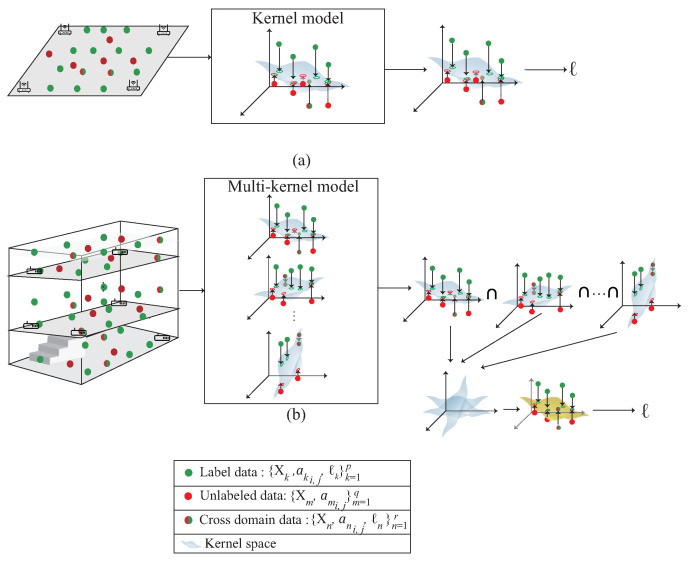
A comparison with (**a**) single kernel learning based on SVM and (**b**) the multi-kernel based semi-supervised learning.

**Figure 4:**
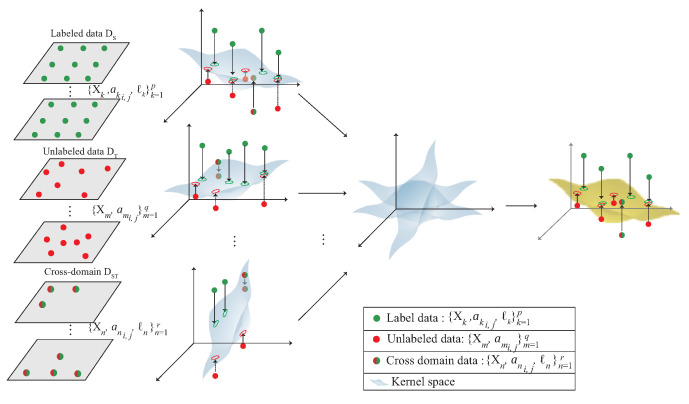
The multi-kernel learning.

Figure 3 shows the difference between the single kernel learning and the multi-kernel learning. The figure is divided into two parts. The upper part represents the single kernel learning; the bottom part is the proposed multi-kernel learning. The proposed scheme is designed for 3D indoor environment compared with the single kernel learning which is designed for 2D plane. By multi-kernel learning, each kernel learns the distribution of own region. According to the signal distribution obtained by mobile device, the expected kernel is the estimated location. The expected kernel is derived from multiple kernels during the multi-kernel optimization phase, which is shown in Figure 4. By using QCQP optimization, each kernel learns the distribution of its own region and fuse all the models to get the distribution of the area of interest.

## 4. A 3D Multi-Kernel Learning Approach for 3D Indoor Localization

**Figure 5:**
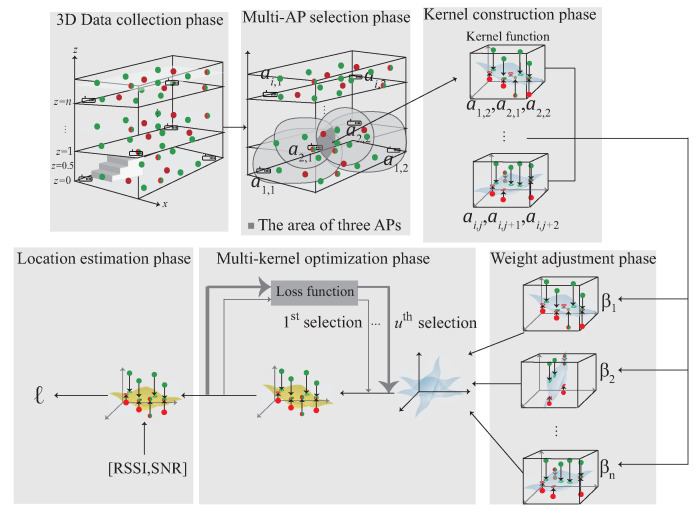
The 3D iterative multi-kernel optimization.

This section proposes a 3D indoor localization scheme based on semi-supervised multi-kernel optimization learning in WiFi network for predicting the unlabeled target with cross-domain data in 3D indoor environment. The notations are defined in Table 1. The system architecture is shown in Figure 5 with six phases. The data acquisition is shown in Figure 4 and divided into the source domain data $D_{S}$ and the target domain data $D_{T}$, which represent the labeled data and the unlabeled data, respectively. The six phases of the proposed scheme are briefly described as follows:

(1) 3D data collection phase: During the data collection stage, the data collected in the 3D environment is divided into $D_{S}$, $D_{T}$ and $D_{ST}$. The $D_{S}$ indicates that the data are in source domain and $D_{T}$ indicates the unlabeled data. $D_{ST}$ is the intersection of $D_{S}$ and $D_{T}$ and can be represented as $D_{ST}\in D_{S}\cap D_{T}$. The collected data are used to choose multiple APs in the next phase.
(2) Multi-AP selection phase: In order to learn the signal distribution generated by each AP, the multi-AP selection phase is proposed. In this phase, according to the signal obtained by the user at the area of interest, select multiple APs near the user. The selected APs form the kernels in the next phase.
(3) 3D Multi-kernel construction phase: In order to find the corresponding signal plane between multiple APs, the multi-kernel construction phase is proposed to solve this problem. In this phase, the selected APs are used to form the kernel model. The data, include $D_{S}$, $D_{T}$ and $D_{ST}$, which located in the area of the intersection between multiple APs is input to the kernel function to form the high-dimensional data. By Mercer’s theorem, the plane between multiple APs is built. The plane is adjusted by the next phase.
(4) Weight adjustment phase: After constructing multiple kernels, the kernel closer to the user is more accurate, therefore, the weight adjustment phase is proposed to determine the weight of each kernel. In this phase, the distance and the SNR between the kernel and the user is measured. If the kernel is closer to the user, the kernel gains a larger weight. The weight of the kernel is optimized in the next phase.
(5) The multi-kernel optimization phase: After giving the weight to each kernel, the area responsible for each kernel is combined according to the weight to form an expected kernel $\;\bar{\;K\;}{\;}_{S}$. In order to reduce the distribution between the source kernel $K_{S}$ and $\;\bar{\;K\;}{\;}_{S}$, the multi-kernel optimization phase is proposed to solve this problem.
(6) Location estimation phase: After multi-kernel optimization phase, the expected kernel $\;\bar{\;K\;}{\;}_{A}$ is constructed and used for location estimation.

### 4.1. 3D data Collection Phase

**Figure 6:**
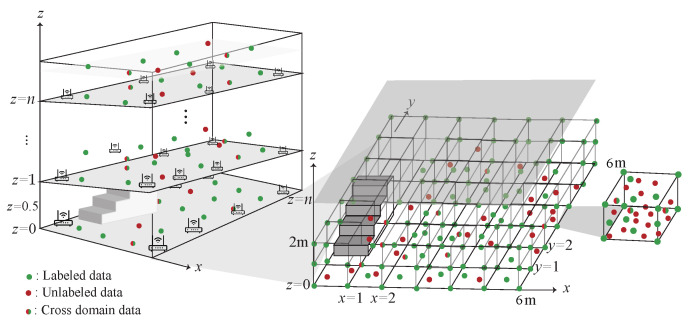
The 3D Data collection phase.

The data collection in the 3D environment is shown in Figure 6. The data are collected at a multi-floor building. Each floor is divided into several cubes and each cube collect training data, which includes RSS, SNR and the corresponding MAC address. The detail of the data collection phase is described as follow:

S1. The data collected in each cube is separated into labeled data ($D_{S}$), unlabeled data ($D_{T}$) and the cross-domain data ($D_{ST}$). $D_{S}={\left\{r_{t},s_{t},a_{i,j},\ell_{i}\right\}}_{t=1}^{p}$ denotes the labeled data which is collected at the eight corners of the cube with $RSS_{t}$, $SNR_{t}$ and the signal collected at the *j*-th AP on the *i*-th floor and the corresponding labeled data $\ell_{t}$, respectively.
S2. $D_{T}={\left\{r_{t},s_{t},a_{i,j}\right\}}_{t=1}^{q}$ denotes the unlabeled data which is collected randomly at each cube with $RSS_{t}$, $SNR_{t}$ and the signal collected at the *j*-th AP on the *i*-th floor, respectively.
S3. $D_{ST}={\left\{r_{t},s_{t},a_{i,j},\ell_{t}\right\}}_{t=1}^{r}$ indicates the cross-domain data with $RSS_{t}$, $SNR_{t}$ and the signal collected at the *j*-th AP on the *i*-th floor, which is the intersection of $D_{S}$ and $D_{T}$ and can be represented as $D_{ST}\in D_{S}\cap D_{T}$.

### 4.2. Multi-AP Selection Phase

**Figure 7:**
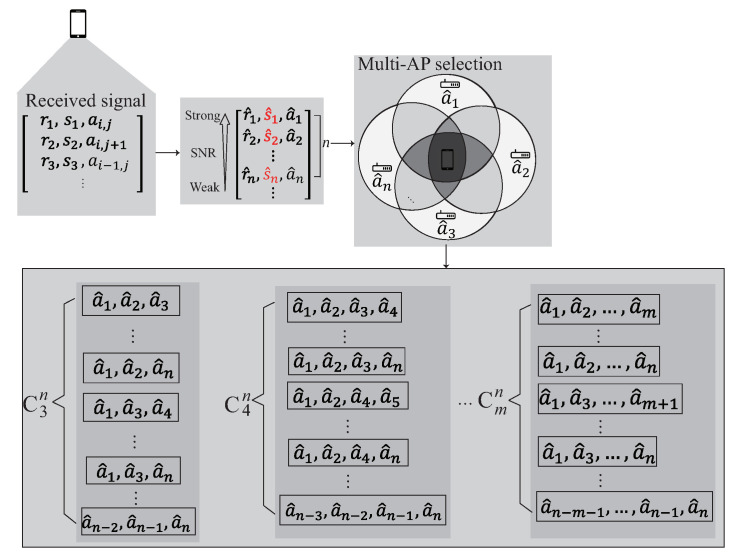
The multi-AP selection phase.

The multi-AP selection phase is shown in Figure 7. Inspired by the existing works [12,20], the ideas of filtering and clustering are adopted in the multi-AP selection phase so as to reduce the training cost and improve the accuracy of localization. In the localization area, the features of the signal including $r_{i}$ and $s_{i}$ often have the data inaccuracy and offset problems which have a considerable impact on the subsequent training of the entire data set. Before multi-AP selection phase, the data with too high deviation value are filtered. The collected data are first sorted according to the deviation of the data in an ascending order. The first *k* data with the lowest deviation is keep the other data are filtered, where *k* is the number of data set candidates. The mean values of $r_{i}$ and $s_{i}$ are computed after removing the outliers. The remaining data are exported for the next step.

The user receives signals at the area of interest. After removing the outliers, the received signal is arranged in descending order according to the value of SNR. Choose the first *n* signals to form the kernel model. The details of the multi-AP selection phase is described as follow:

S1. The collected signals are arranged in descending order according to the value of SNR and stored into *Q*. *Q* can be represented as $Q=\{\left[r_{1},s_{1},a_{1}\right],\left[r_{2},s_{2},a_{2}\right],\left[r_{3},s_{3},a_{3}\right],\cdots$, $\left[r_{k},s_{k},a_{k}]\right\}$, where *k* is the number of data set candidates which can be collected at the reference point.
S2. According to the signals stored in *Q*, choose the first *n* signals from *Q*, where $Q=\{\left[{\hat{r}}_{1},{\hat{s}}_{1},{\hat{a}}_{1}\right],[{\hat{r}}_{2},{\hat{s}}_{2}$, ${\hat{a}}_{2}],\cdots ,\left[{\hat{r}}_{n},{\hat{s}}_{n},{\hat{a}}_{n}\right]\}$ is the *n* best signals which can be heard at the reference point. The intersection between each AP’s transmission range have different signal distribution. In order to learn the signal distribution generated by each AP, each element in *Q* is permutation to select multiple APs whose signal is within the *n* best signals and stored in cluster *C*.
S3. The cluster *C* can be represented as $C=\{[{\underset{︸}{{\hat{a}}_{1},{\hat{a}}_{2}],\cdots }}_{C_{2}^{n}},[{\underset{︸}{{\hat{a}}_{1},{\hat{a}}_{2},{\hat{a}}_{3}],\cdots }}_{C_{3}^{n}},\cdots ,$$\left[{\underset{︸}{{\hat{a}}_{1},{\hat{a}}_{2},\cdots ,],\cdots ,{\hat{a}}_{m}}}_{C_{m}^{n}}\right\}$, where *m* represents the number of AP groups. In the following phase, each component of the cluster is operated separately to form the kernel.

### 4.3. Multi-Kernel Construction Phase

In the multi-kernel construction phase, in order to form the kernel, the data, including $D_{S}$, $D_{T}$ and $D_{ST}$, is mapped to high dimension by kernel function. The following lemma proves that the data do not disappear when it is mapped to high dimension and can form the kernel space by Mercer’s theorem.

**Mercer’s Theorem**  [21]:*If k(x,y) is a kernel function, then k(x,y) must satisfy the Mercer’s condition, that is $\sum_{i=1}^{n}\sum_{j=1}^{n}k\left(x_{i},y_{i}\right)\geq 0$, ∀x∀y∈R.*

**Lemma** **1.** *The RBF kernel is $K_{R}\left(x,y\right)=exp\left(\frac{-d{\left(x,y\right)}^{2}}{2l^{2}}\right)$, where l>0 is the parameter and d(x,y) is the Euclidean distance between x and y in high dimension space. The chi-squared kernel is $K_{C}=exp\left(-\gamma \sum_{i}\frac{{\left(x\left[i\right]-y\left[i\right]\right)}^{2}}{x[i]+y[i]}\right)$, where γ∈[0,1] is the parameter. Let data $\{\left[r_{1},s_{1},a_{1}\right],\left[r_{2},s_{2},a_{2}\right],\cdots ,$$\left[r_{m},s_{m},a_{m}\right],\left[r_{m+1},s_{m+1}\right],\left[r_{m+2},s_{m+2}\right],\cdots ,\left[r_{n},s_{n}\right],\left[r_{n+1},s_{n+1},a_{n+1}\right][r_{n+2},s_{n+2},$$a_{n+2}],\cdots ,$$\left[r_{k},s_{k},a_{k}]\right\}$ denote the $D_{S}$, $D_{T}$, and $D_{ST}$, respectively, where $D_{S}\in R^{m}$,$D_{T}\in R^{n-m}$ and $D_{ST}\in R^{k-n}$. The $D_{S}$, $D_{T}$ and $D_{ST}$ can be mapped to high dimension by RBF kernel to form the kernel and can also be mapped by chi-squared kernel to form the kernel.*

**Proof**  According to the definition of kernel function, there is a function that satisfies k(x,y)=⟨Φ(x),Φ(y)⟩ for all data, the k(x,y) is a kernel function. By Mercer’s theorem, if k(x,y) is a kernel function, then k(x,y) must satisfy the Mercer’s condition, that is $\sum_{i=1}^{n}\sum_{j=1}^{n}k\left(x_{i},y_{j}\right)\geq 0,\forall x\forall y\in R.$ The RBF kernel is $K_{R}\left(x,y\right)=exp\left(\frac{-d{\left(x,y\right)}^{2}}{2l^{2}}\right)$, by Taylor series $e^{x}=1+\sum_{n=1}^{\infty }1+x+\frac{x^{2}}{2!}+\frac{x^{3}}{3!}+\cdots +\frac{x^{n}}{n!}=\lim_{x\to \infty }{\left(1+\frac{x}{n}\right)}^{n}\approx 2.7^{x}$. The RBF kernel can be rewrite as $K_{R}\left(x,y\right)=2.7^{\frac{-d{\left(x,y\right)}^{2}}{2l^{2}}}$, where $K_{R}\left(x,y\right)>0$, ∀x∀y∈R, and satisfies the Mercer’s condition. If $K_{R}\left(x,y\right)>0$, ∀x∀y∈R, this condition means that the data do not disappear ∀x∀y∈R when it is mapped to high dimension and can form the kernel. The chi-squared kernel is $K_{C}=exp\left(-\gamma \sum_{i}\frac{{\left(x\left[i\right]-y\left[i\right]\right)}^{2}}{x[i]+y[i]}\right)$ and can be rewrite as $K_{C}=2.7^{-\gamma \sum_{i}\frac{{\left(x\left[i\right]-y\left[i\right]\right)}^{2}}{x[i]+y[i]}}$. Under the condition of chi-squared kernel, the $K_{c}\left(x,y\right)>0$, $\forall x\forall y\in R^{+}$ and satisfies the Mercer’s condition. If $K_{c}\left(x,y\right)>0$, $\forall x\forall y\in R^{+}$, this condition means that the data do not disappear $\forall x\forall y\in R^{+}$ when it is mapped to high dimension and can form the kernel space. Because $K_{R}\left(x,y\right)>0$ and $K_{c}\left(x,y\right)>0$, the $D_{S}$, $D_{T}$ and $D_{ST}$ can be mapped to high dimensions by RBF kernel to form the kernel, and can also be mapped by chi-squared kernel to form the kernel without lost data.    □

**Figure 8:**
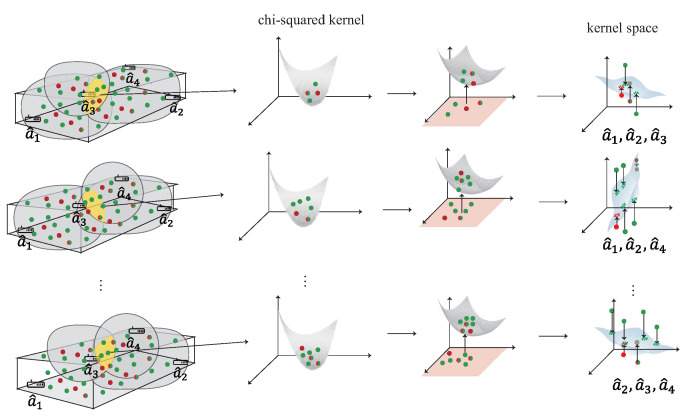
The multi-kernel construction phase.

The kernel learning represents the domain-invariant kernel by matching the source distribution and the target distribution to generate the kernel Hilbert space. In multi-AP selection phase, each group of the cluster’s data, including $D_{S}$, $D_{T}$ and $D_{ST}$, represent the intersection of the area formed by multiple APs. For example, if the intersection area is formed by two APs $\;\bar{\;a\;}{\;}_{1}$ and $\;\bar{\;a\;}{\;}_{2}$, the data must have signal heard by $\;\bar{\;a\;}{\;}_{1}$, $\;\bar{\;a\;}{\;}_{2}$. Each group of cluster’s data is operated separately to form the kernel in this phase. The detailed of multi-kernel construction phase is shown as Figure 8 and is described as follow:

S1. The first step is to calculate the source kernel $K_{S}$ and the target kernel $K_{T}$ using the kernel function, such as chi-squared kernel $K\left(x,y\right)=exp\left(-\gamma \sum_{i}\frac{{\left(x\left[i\right]-y\left[i\right]\right)}^{2}}{x[i]+y[i]}\right)$. The cross-domain kernel $K_{ST}$, from the common area between $K_{S}$ and $K_{T}$, are also using the chi-squared kernel.
S2. In order to evaluate the distribution difference between $K_{S}$ and $K_{T}$ in the space of Hilbert, the second step is to compute the difference of distribution between the source kernel and the target kernel. However, since $K_{S}$ and $K_{T}$ have distinct dimensions, that is $\;\bar{\;K\;}{\;}_{S}\in R^{l_{S}\times l_{S}}$ and $K_{T}\in R^{l_{T}\times l_{T}}$, the difference between $K_{S}$ and $K_{T}$ cannot be directly estimated. In order to solve the problem of different dimensions, an extrapolated kernel $\;\bar{\;K\;}{\;}_{S}\in R^{l_{S}\times l_{S}}$ is constructed using the eigensystem $\left\{\Phi_{T},\lambda_{T}\right\}$. The target eigenvector matrix $\Phi_{T}$ and the target eigenvalue matrix $\lambda_{T}$ can be constructed using the standard problem of eigenvalue $K_{T}\Phi_{T}=\Phi_{T}\lambda_{T}$.
S3. After calculated eigensystem and the cross-domain kernel $K_{ST}$, the extrapolated source kernel $K_{S}$ of the eigenvector matrix is calculated by the Mercer’s theorem as $\;\bar{\;\Phi_{S}\;}\;\approx K_{ST}\Phi_{T}\lambda_{T}^{-1}$.
S4. After S3, the new source kernel $\;\bar{\;K\;}{\;}_{S}$ is constructed from the eigenvectors of the target kernel, where $\;\bar{\;K\;}{\;}_{S}=\;\bar{\;\Phi \;}{\;}_{S}\lambda_{T}\Phi_{S}^{-1}$. In the next phase, each kernel is given a weight according to the distance and the SNR value between the user and the kernel.

The pseudo code of the multi-kernel construction phase is shown in Algorithm 1.
**Algorithm 1:** The multi-kernel construction phase**Input:** The dataset of $D_{S_{i}},D_{T_{i}}$ and $D_{ST_{i}}$ derived in the multi-AP selection phase$D_{S_{i}}=$${\left[\begin{array}{c} \left[{\hat{r}}_{1},{\hat{s}}_{1},{\hat{a}}_{1},\ell_{1}\right],\left[{\hat{r}}_{2},{\hat{s}}_{2},{\hat{a}}_{2},\ell_{2}\right],\cdots ,\left[r_{m},s_{m},{\hat{a}}_{m},\ell_{m}\right] \end{array}\right.}_{i}$$D_{T_{i}}=$${\left[\begin{array}{c} \left[{\hat{r}}_{1},{\hat{s}}_{1},{\hat{a}}_{1}\right],\left[{\hat{r}}_{2},{\hat{s}}_{2},{\hat{a}}_{2}\right],\cdots ,\left[{\hat{r}}_{n},{\hat{s}}_{n},{\hat{a}}_{n}\right] \end{array}\right.}_{i}$$D_{ST_{i}}=$${\left[\begin{array}{c} \left[{\hat{r}}_{1},{\hat{s}}_{1},{\hat{a}}_{1},\ell_{1}\right],\left[{\hat{r}}_{2},{\hat{s}}_{2},{\hat{a}}_{2},\ell_{2}\right],\cdots ,\left[{\hat{r}}_{d},{\hat{s}}_{d},{\hat{a}}_{d},\ell_{d}\right] \end{array}\right.}_{i}$**Output:** The multiple domain-invariant kernel $K=\{\;\bar{\;K\;}{\;}_{S_{1}},\;\bar{\;K\;}{\;}_{S_{2}},\cdots ,\;\bar{\;K\;}{\;}_{S_{u}}\}$, each $\;\bar{\;K\;}{\;}_{S_{i}}$ represent the kernel model form by $D_{S_{i}},D_{T_{i}}$ and $D_{ST_{i}}$, where $u=\sum_{j=3}^{m}C_{j}^{n}$*K*=*∅***for***j=3 to m***do**
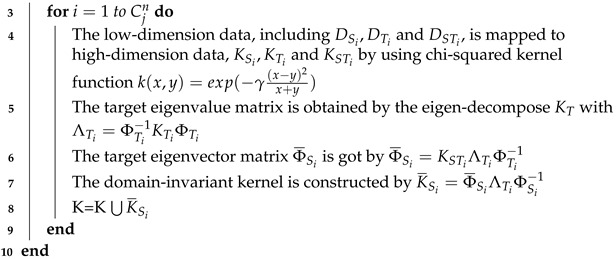



### 4.4. The Weight Adjustment Phase

**Figure 9:**
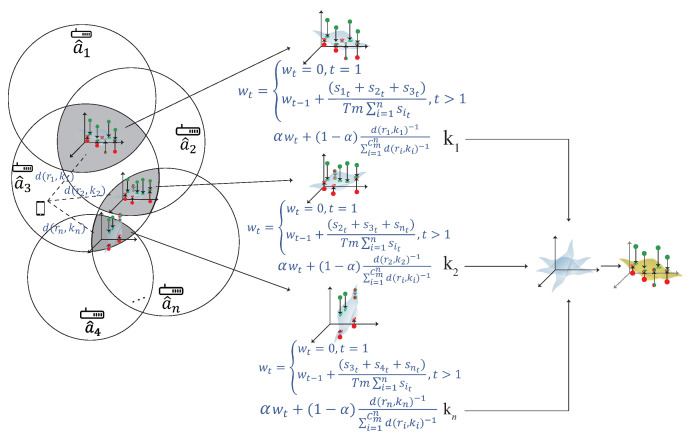
The weight adjustment phase.

The weight adjustment phase is shown in Figure 9. After constructing multiple kernels, the kernel closer to the user have higher accuracy. In order to find the relationship between the kernel and the user, the distance is measured between the kernel and the user. If the kernel is closer to the user, the kernel has a larger weight. According to the signal that user received, the distance between each AP can be calculated by the signal strength, the detailed of the weight adjustment phase is described as follow:

S1. The distance between the user and AP can be calculated by $d\left(r,\hat{a}\right)=10^{\frac{P-r}{10n}}$, where *P* is the measured power from $\hat{a}$, *n* is the environment factor.
S2. The distance between the user and the kernel can be calculated by $f_{d}\left(R,k\right)$, where *R* represent the corresponding RSS to form the kernel *k*. For example, if kernel *k* is formed by ${\hat{a}}_{1},{\hat{a}}_{2},{\hat{a}}_{3}$, then $f_{d}\left(R,k\right)=\frac{d\left(r_{1},{\hat{a}}_{1}\right)+d\left(r_{2},{\hat{a}}_{2}\right)+d\left(r_{3},{\hat{a}}_{3}\right)}{3}$, where $r_{1}$,$r_{2}$ and $r_{3}$ denotes the RSS that the user received from ${\hat{a}}_{1},{\hat{a}}_{2}$ and ${\hat{a}}_{3}$, respectively.
S3. The SNR signal strength can be calculated by $w_{t}=$ $\left\{\begin{array}{c} w_{t}=0,t=1\\ w_{t-1}+\frac{f_{s}\left(S,K\right)}{Tm\sum_{i=1}^{n}S_{i_{t}}},t>1 \end{array}\right.$, where *T* denotes the length of time, $f_{s}\left(S,K\right)$ is the sum of the corresponding SNR signal to form the kernel model *k*, and *m* represents the number of AP. For example, if kernel model *k* is formed by $\;\bar{\;a\;}{\;}_{1}$,$\;\bar{\;a\;}{\;}_{2}$,$\;\bar{\;a\;}{\;}_{3}$, then $w_{t}=w_{t-1}+\frac{s_{1_{t}}+s_{2_{t}}+s_{3_{t}}}{Tm\sum_{i=1}^{n}S_{i_{t}}}$, where $s_{1_{t}}$, $s_{2_{t}}$ and $s_{3_{t}}$ denotes the SNR that the user received from ${\hat{a}}_{1},{\hat{a}}_{2}$ and ${\hat{a}}_{3}$.
S4. The weight of each kernel can be determined by $\alpha w_{t}+\left(1-\alpha \right)\frac{f{\left(R,k_{i}\right)}^{-1}}{\sum_{i=1}^{C_{m}^{n}}f{\left(R,k_{i}\right)}^{-1}}$, where α∈[0,1]. If the kernel is closer to the user, the kernel has a larger weight and if the kernel is farther to the user, the kernel has a smaller weight. In order to formalize the distribution discrepancy between the new source kernel $\;\bar{\;K\;}{\;}_{S}$ and the ground truth source kernel $K_{S}$, the multi-kernel optimization is used in the next phase to solve this problem.

The pseudo code of the weight adjustment phase is shown in Algorithm 2.
**Algorithm 2:** The weight adjustment phase**Input:** The multiple domain-invariant kernel $K=\{\;\bar{\;K\;}{\;}_{S_{1}},\;\bar{\;K\;}{\;}_{S_{2}},\cdots ,\;\bar{\;K\;}{\;}_{S_{u}}\}$ construct by Algorithm 1**Output:** The weight of each domain-invariant kernel $P=\{\beta_{1}\;\bar{\;K\;}{\;}_{S_{1}},\beta_{2}\;\bar{\;K\;}{\;}_{S_{2}},\cdots ,\beta_{u}\;\bar{\;K\;}{\;}_{S_{u}}\}$*P*=*∅***foreach***kernel $\;\bar{\;K\;}{\;}_{S_{i}}$ in K***do**
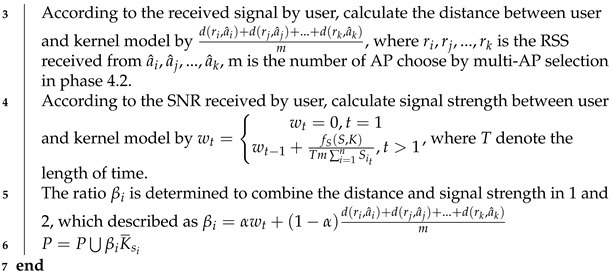


### 4.5. The Multi-Kernel Optimization Learning Phase

**Figure 10:**
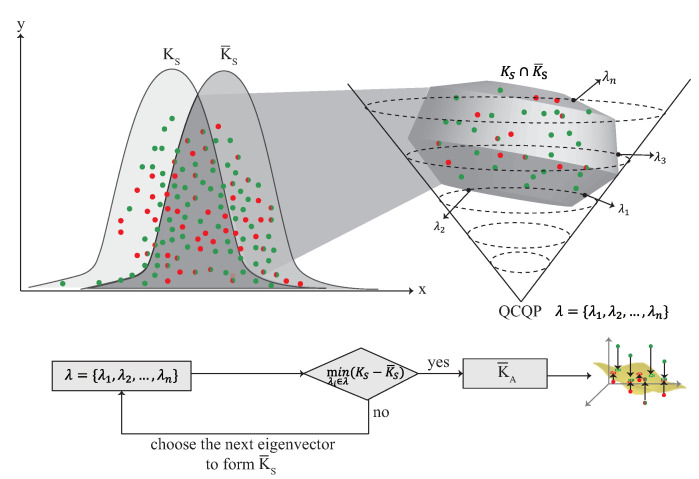
The multi-kernel optimization phase.

After the weight adjustment phase, each kernel model has its own weight. In multi-kernel optimization phase, each kernel is adjusted by the QCQP optimization as shown in Figure 10. $K_{C}$ is the source domain and $\;\bar{\;K\;}{\;}_{C}$ is constructed by $K_{C}$ using Mercer’s theorem, where $C=S_{1},S_{2},\cdots ,S_{u}$. Each kernel can be represented as the distribution of the localization area, and is combined according to the weight to form a combined kernel $\;\bar{\;K\;}{\;}_{S}$, where $\;\bar{\;K\;}{\;}_{S}=\beta_{1}\;\bar{\;K\;}{\;}_{S_{1}}+\beta_{2}\;\bar{\;K\;}{\;}_{S_{2}}+\cdots +\beta_{u}\;\bar{\;K\;}{\;}_{S_{u}}$. The $K_{S}$ is the source domain combined with the weight which is denoted as $K_{S}=\beta_{1}K_{S_{1}}+\beta_{2}K_{S_{2}}+\cdots +\beta_{u}K_{S_{u}}$. In order to minimize the difference between $\;\bar{\;K\;}{\;}_{S}$ and $K_{S}$, the QCQP optimization is used. The detail of the multi-kernel optimization phase is described as follow:

S1. In order to minimize the difference between $K_{S}$ and $\;\bar{\;K\;}{\;}_{S}$, the QCQP optimization is used which is described as $\min_{\lambda_{i}\in \lambda }{\left(\;\bar{\;K\;}{\;}_{S}-K_{S}\right)}^{2}$, where $\lambda =\{\lambda_{1},\lambda_{2},\cdots ,\lambda_{n}\}$ are the *n* nonnegative eigen-spectrum parameters.
S2. The QCQP optimization is used to derive the intersection between $K_{S}$ and $\;\bar{\;K\;}{\;}_{S}$ in the high dimension. The crossover point in $K_{S}\cap \;\bar{\;K\;}{\;}_{S}$ is λ found by QCQP optimization, where i∈1,2,⋯,n.
S3. The goal of the multi-kernel optimization phase is to find a $\lambda_{i}$ that can minimize the difference between $K_{S}$ and $\;\bar{\;K\;}{\;}_{S}$. In this step, $K_{C}^{\prime }$ is calculated by $K_{C}^{\prime }=\Phi_{C}\lambda_{i}\Phi_{C}^{T}$, where $\Phi_{C}$ is the eigenvector matrix and $\lambda_{i}$ is the eigenvalue matrix. $\;\bar{\;K\;}{\;}_{A}$ is constructed with $\lambda_{i}$ which has the minimized distribution of $K_{S}\cap \;\bar{\;K\;}{\;}_{S}$.

### 4.6. Location Estimation Phase

After the multi-kernel optimization learning, $\lambda_{i}$ is found and forms $\;\bar{\;K\;}{\;}_{A}$ with the minimized difference between $K_{S}$ and $\;\bar{\;K\;}{\;}_{S}$. Finally, $\;\bar{\;K\;}{\;}_{A}$ is used to estimate the location with the signal received by the user.

S1. After $\;\bar{\;K\;}{\;}_{A}$ is constructed in multi-kernel optimization learning, the user’s location $\ell_{t}$ can be estimated by $\;\bar{\;K\;}{\;}_{A}$ according to the signal received by user.
S2. The user’s location $\ell_{t}$ can be denoted as $\ell_{t}=\;\bar{\;K\;}{\;}_{A}\left(\left\{\left[r_{1},s_{1},a_{1}\right],\left[r_{2},s_{2},a_{2}\right],\cdots ,\left[r_{n},s_{n},a_{n}\right]\right\}\right)$, where $\left\{\left[r_{1},s_{1},a_{1}\right],\left[r_{2},s_{2},a_{2}\right],\cdots ,\left[r_{n},s_{n},a_{n}\right]\right\}$ represents the *n* best signals received by the user.

## 5. Experiment Results

The experiments are performed in the Elytone building of National Taipei University. 8 APs are deployed in a 20 m × 20 m plane with the height of 2 m to construct the multiple kernels on each floor. In total, there are 24 APs deployed in three consecutive floors. User can receive signals from APs on the upper and lower floors. The cube size is 1 m × 1 m × 1 m. More than 1000 samples are collected at each cube. After filtering, 800 samples with lower deviation are used for training. The proportion of the labeled data to unlabeled data and cross-domain data is 1:2:1. Hence, the proportion of the labeled data is 25%. To verify the efficiency of the proposed scheme, seven schemes, namely, the proposed multi-kernel scheme with chi-squared as the kernel function (denoted as CMK) and RBF as the kernel function (denoted as RMK), the single-kernel scheme with chi-squared as the kernel function (denoted as CSK) and RBF as the kernel function (denoted as RSK), the hybrid-kernel scheme with the hybrid of RBF and chi-squared (denoted as RBF + chi-squared) and the hybrid of RBF and polynomial (denoted as RBF + polynomial), and the scheme using multiple DNN models (denoted as multi-DNN) are experimented and compared in this paper. The performance metrics to be observed are defined as follows.

- Localization error: The average difference between the real location and the estimated location.
- Localization accuracy: The ratio that the estimated location is located at the same cube as the real location.
- Learning time: The time it takes to form each kernel.

### 5.1. Localization Error

**Figure 11:**
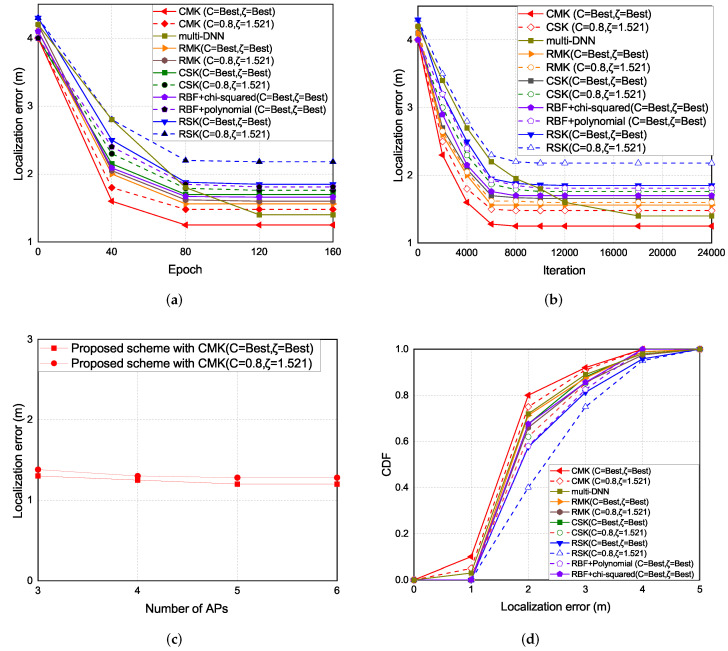
(**a**) Localization error vs. epoch.(**b**) Localization error vs. iteration. (**c**) Localization error vs. number of selected APs with epoch 80. (**d**) CDF vs. localization error.

The experiment results of the localization error are shown in Figure 11. As the epoch increases the localization error decreases. The localization error of the CMK scheme is the lowest, followed by the multi-DNN, RMK, RBF + chi-squared, CSK, RBF+polynomial, and the RSK schemes. Overall, the schemes with chi-squared as the kernel function performs better than the schemes with RBF as the kernel function and the proposed multi-kernel schemes performs better than the hybrid-kernel and single-kernel schemes because we create multiple kernels and try to get the optimal expected kernel and thus the proposed multi-kernel scheme can reduce the localization error and enhance the localization accuracy.

The experiment results of the localization error under each iterations and epoch are shown in Figure 11a,b. There are two parameters in the experimental results, the *C* is the regularization parameter. The intensity of normalization is inversely proportional to *C*. ζ is the error caused by how much data are allowed. The greater the value is, the greater the error is allowed. In the single kernel part, the chi-squared single kernel (CSK) scheme is better than the RBF single kernel (RSK) scheme, because of the different distribution in the RBF kernel and chi-squared kernel. The distribution of the RBF kernel and chi-squared kernel are shown in Figure 8. The color with dark part is the place where the data are gathered. In the RBF kernel, the place where the data are gathered is more separated than the chi-squared kernel according to the similarity of the signal received by data collection in 3D environment, which leads to higher localization error compared with the chi-squared kernel. In the multi-kernel part, the hybrid kernel (RBF+polynomial, RBF+chi-squared) schemes generally are better than the single kernel schemes, in order to reduce the localization error, the 3D multi-kernel learning scheme is proposed which improves the shortcomings of the singe kernel and hybrid kernel schemes. The results show that the chi-squared multi-kernel (CMK) scheme is better than the RBF multi-kernel (RMK) and the single kernel schemes in the 3D localization environment. In multi-DNN, by creating multiple DNN models to cover the localization area, the location of the user can be calculated by using probability that the phone is located at a given location. Since multi-DNN needs to adjust the weight of each neuron, the convergence speed is slower than that of the multi-kernel scheme. Figure 11c presents the localization error with number of APs which are selected in the multi-AP selection phase. Basically, the more APs are chosen, the lower localization error will be. However, if too many APs are selected, the complexity of the 3D multi-kernel construction will increase. Figure 11d presents the CDF of the localization error.

### 5.2. Localization Accuracy

**Figure 12:**
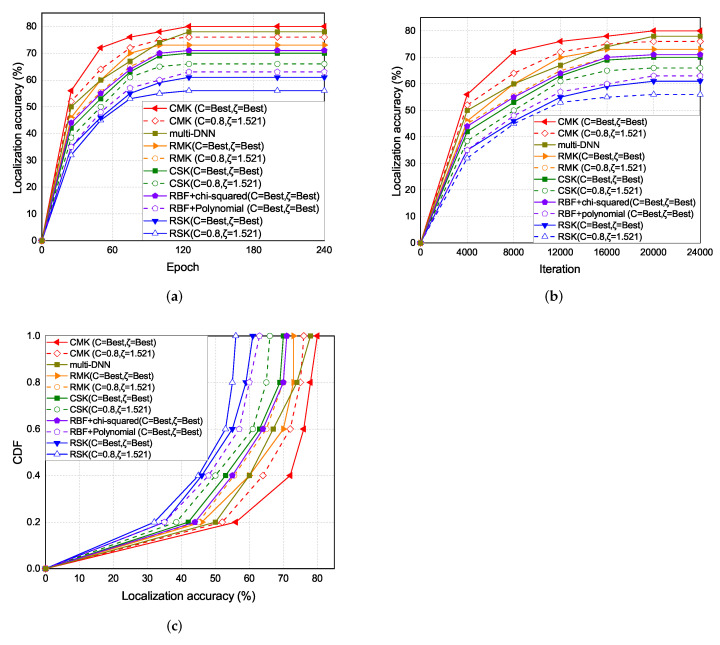
(**a**) Localization accuracy vs. epoch. (**b**) Localization accuracy vs. iteration. (**c**) Localization error vs number of selected APs with epoch 80.

The experiment results of the localization accuracy are shown in Figure 12. As the epoch increases the localization accuracy also increases. The localization accuracy of the CMK scheme is the highest, followed by the multi-DNN, RMK, RBF+chi-squared, CSK, RBF+polynomial, and the RSK schemes.

The experiment results of the localization accuracy under each iterations and epoch are shown in Figure 12a,b. In the single kernel part, the accuracy of CSK is better than RSK because of the different distribution in RBF kernel and chi-squared kernel in Figure 8. In the multi-kernel part, the accuracy of CMK and hybrid kernel is also better than RMK.

### 5.3. Time Cost

**Figure 13:**
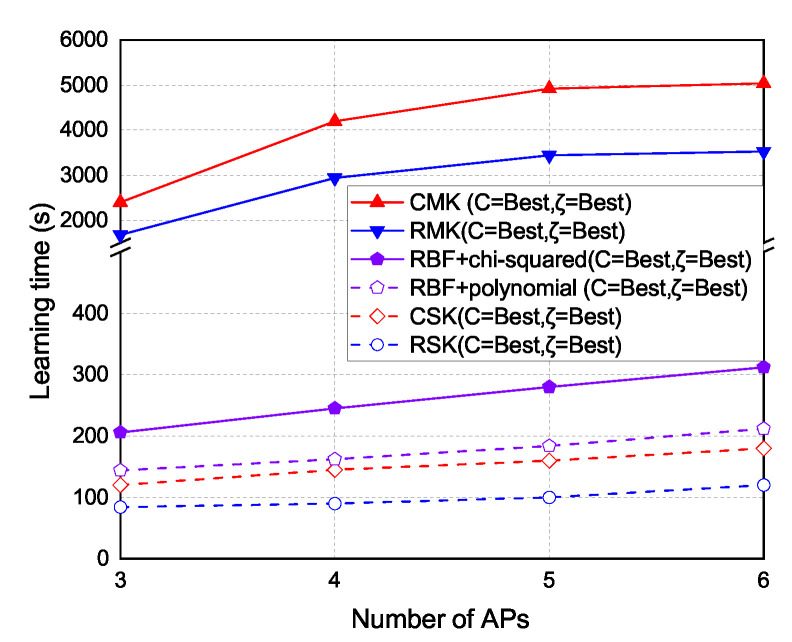
Time cost vs. number of selected APs.

The experiment results of the learning time under different number of APs are shown in Figure 13. As the number of APs increases, the time to form the kernel models also increases. The learning time of RSK and CSK schemes is the lowest because the single-kernel schemes only need to form a single kernel. The hybrid kernel schemes take more time than RSK and CSK, because the hybrid kernel schemes need to form two kernels. The learning time of RMK and CMK schemes is the highest because the multi-kernel schemes need to form multiple kernels.

The experiment results of time cost under each number of APs are shown in Figure 13. Basically, if too many APs are selected, the time to form kernel model will increase. In the single kernel part, the time cost of RSK and CSK are the least because they use a single kernel to cover the localization area. In the multi-kernel part, the hybrid kernel takes more times than RSK and CSK, because the hybrid kernel uses two kernels to cover the localization area. In RMK and CMK, the time cost to form multiple kernels are the most, because the multi-kernel scheme uses multiple kernels to cover the localization area.

### 5.4. Discussions

According to the above experiment results, the proposed multi-kernel scheme can achieve the lowest localization error and the highest localization accuracy because through the multi-AP selection, 3D multi-kernel construction, weight adjustment, and the multi-kernel optimization phases, the proposed multi-kernel scheme can derive the best expected kernel through multiple kernels constructed by the signals collected from the selected APs and thus the proposed localization scheme can estimate the location more accurately and hence reduce the localization error. The CMK scheme performs better than the RBF scheme because the signal obtained by users with short distance movement is not obviously different after mapped to high-dimension by RBF kernel which leads to higher localization error as opposed to the chi-squared kernel.

The multi-DNN scheme performs the second best in terms of localization error and localization accuracy. However, the multi-DNN scheme needs to adjust the weight of each neuron, the convergence speed is slower than that of the multi-kernel scheme. Besides, the multi-DNN scheme needs to collect more sample data.

The time cost of the proposed multi-kernel scheme is higher than the other kernel-based scheme, because proposed multi-kernel scheme needs to construct multiple kernels, adjust weight for each kernel, and optimize the expected kernel and hence the time cost of the proposed scheme is the highest. Basically, the more the kernels are constructed, the more the time cost becomes. The time cost is also proportional to the number of selected APs.

## 6. Conclusions

In this paper, we present a new 3D localization scheme which is able to construct multiple kernels for 3D indoor localization. Compared with the traditional kernel learning scheme, we create multiple kernels to improve the localization accuracy. The proposed scheme considers multiple parameters and learns the signal’s distribution generated by each AP in each cube from the source domain and transfer to the target domain. Finally, we provide the experiment results and show that the proposed scheme performs better than the multi-DNN scheme and the existing kernel-based localization schemes in terms of localization accuracy and error in the 3D indoor environment.

## Figures and Tables

**Table 1:** Notation table.

$D_{S}$	The source domain
$D_{T}$	The target domain
$D_{ST}$	The cross-domain
$\left\{r_{m},s_{m},a_{i,j}\right\}$	The three tuples, which represent the *m*-th RSSI, SNR signal and received from the *j*-th AP on the *i*-th floor
$K_{S}$	The source kernel
$K_{T}$	The target kernel
$K_{ST}$	The cross domain kernel
*C*	The cluster which AP is selected
$\;\bar{\;K\;}{\;}_{S}$	The extrapolated source kernel
$\lambda_{T}$	The estimated location through 3D multi-kernel learning phase
*ℓ*	The target’s eigenvalue
$\beta_{1},\beta_{2},\cdots ,\beta_{k}$	The weight of each kernel

## Data Availability

Data sharing is not applicable to this article.
